# Supplementary material for: Predictors and experiences of seeking abortion services from pharmacies in Nepal
Source: PLOS Glob Public Health. 2024 May 9;4(5):e0003144. doi: 10.1371/journal.pgph.0003144 (PMC11081245; doi:10.1371/journal.pgph.0003144)
Supplement: Attachment — Submitted filename: Plos_response to Reviewers_16Feb24.docx [file pgph.0003144.s002.docx]

February 16, 2024

To the editors and reviewers

Thank you for your feedback on our paper, now titled “Predictors and experiences of seeking abortion services from pharmacies in Nepal” We greatly appreciated the reviewer feedback and have provided point by point responses below

Please let us know if you have further questions

Best

Nadia Diamond-Smith, on behalf of my co-authors

Reviewer #1: 01) Current needs to be improved. The term "pharmacy abortion denial" might be rephrased for smoother readability. Consider using terms like "pharmacy-assisted abortion denial" for precision and coherence. While the current title focuses on denial, the positive outcomes and experiences of those who successfully receive pharmacy-assisted abortion services are not emphasized. Including a mention of both denial predictors and positive experiences could provide a more balanced perspective.
Ex:- "Beyond Legal Boundaries: Exploring Pharmacy-Assisted Abortion in Nepal and Predictors of Denial"
"Pharmacists as Gatekeepers: Examining Experiences and Denial Predictors of Abortion Services in Nepal"

Thank you for these suggestions. We have changed the title to “Predictors and experiences of seeking abortion services from pharmacies in Nepal”

02) Line 142 - Could the authors provide clarity on the rationale behind selecting Chitwan and Rupendehi districts for the study? Was the selection based on purposive sampling to ensure a diverse representation, or was it more of a convenience sampling approach? Elaborating on the criteria for district selection would enhance the reader's understanding of the study's context and strengthen the methodological transparency.

We have added the following “These two districts are in distinct provinces and were selected purposively to have diverse representation, availability of abortion data, availability list of pharmacies from the previous study, built in trust form previous work and willingness to support the study.”

03) "On line 158, the authors specify that the study included women aged 17-45 years. Given that the typical reproductive lifespan of women extends up to 49 years, it would be beneficial for the authors to provide a rationale for the chosen age range. Clarifying the reasons behind this decision would help readers understand the scope of the study and any potential implications for generalizability to the broader reproductive-age population."

Apologies, we had IRB approval to recruit anyone over aged 15, the sample just ended up being women 17-45, but was not restricted to that. We have edited accordingly.


04) On line 191, the authors state that they created an objective measure of socioeconomic status using asset variables to determine participants' wealth quintile. To ensure the validity and reliability of the measurement scale, could the authors provide information on whether a standardized measurement scale, validated in the context of Nepal, was employed? If so, including references to the validated measurement scale would strengthen the methodological rigor and contextual relevance of the socioeconomic status assessment in this study.

We used the standard set of items in the Nepal Demographic and Health Surveys, we have added the reference.

05) On line 208, the authors mention that differences between groups were determined using bivariate analysis and t tests. Considering the potential impact of non-normality on the validity of t tests, it would be valuable for the authors to clarify whether normality assumptions were tested. If the data did not meet the normality assumption, a mention of the use of nonparametric tests for rank data, which are more suitable in such cases, would enhance the robustness of the statistical analysis.

Thank you for this comment. We had originally described the age variable as continuous data within table 2 illustrating sociodemographic characteristics, thus using a t-test to determine differences between the age of women who had received medical abortion pills compared with women denied abortion pills. However, we since re-coded this variable to categorical, rendering the t-test inapplicable. Instead the fisher’s exact test was used to determine differences between the women by age categories. As such, we have adjusted the language in text to say, “Differences between groups were determined through bivariate analysis using fisher’s exact or chi2 tests where appropriate.”

06) I could not locate information on the sample size and the procedure followed for sample size calculation in the manuscript. To ensure transparency and interpretability of the study findings, it is crucial to include details regarding the rationale behind the chosen sample size and the method used for its determination. Please provide this information in the manuscript to enhance the overall methodological clarity and robustness of the study.

Since this was an add on to a larger study of women recruited from clinics, and we were not fully sure if women would be willing to talk about this practice since it is illegal, our sample was determined by budget and client flow. We have added the following in the methods. “The sample size was based on budget and time restrictions, and because it was uncertain if women would be willing to participate given the sensitive nature of receiving medication abortion illegally from a pharmacist, it was not powered to detect differences between groups (clinic/pharmacy; denied/received).”

07) In Table 1, the section on 'Reasons for denial' appears to have limited variability, with only one respondent categorizing for most options. This raises concerns about the informativeness of the data in this table. It may be beneficial to reconsider the presentation of this information in other form.

Thank you for this comment, we agree that the main point is that most women were denied because of the gestational age and the other responses have small Ns. We considered grouping the different other categories together, however, felt that they were fairly different and it was somewhat interesting to see the other types of reasons listed. Therefore, we ask to keep it for its descriptive/qualitative value, if not quantitative value.

08) In Table 2, it would be valuable to enhance the presentation of the baseline sociodemographic characteristics by adding an additional column to include p-values and the corresponding test statistics. This addition would offer readers a clear understanding of the type of test used and the statistical significance of differences between women who received and were denied pharmacy abortion services.

Thank you for this comment. We have added the additional column to include the test statistic and corresponding p-value.

09) In Table 3, the section on 'Reasons for choosing pharmacy for abortion services' could be simplicity. Considering the limited frequency (one or two) for the last four categories, it may be beneficial to merge them into a single category labeled 'Other.'

Thank you for this comment, we have merged the last four categories as suggested to one category for “Other”.

10) To improve the readability of Table 6, it is suggested to merge the second column (Odds Ratio) and the third column (95% Confidence Interval of the Odds Ratio). This can be achieved by presenting the Odds Ratio and its corresponding confidence interval in a single column as 'OR (95% CI),' for example: '0.85 (0.72-0.99)' for age.

Thank you for this comment, we have merged the second column (OR) with the third column (95% CI) to improve readability as suggested.

11) While the study provides valuable insights, it would be beneficial to include a dedicated section discussing the limitations. Consider addressing factors such as the sample size, the number of pharmacies selected, and the adopted methodology.

We have expanded and clarified the limitations section “This study has strengths and limitations. Its value is its unique approach in recruiting women at the time of abortion seeking, and following them longitudinally, thus eliminating some potential biases due to recall and sensitivities reporting abortion seeking or receipt. Limitations include the small sample size and fact that we recruited from only four pharmacies, in only two districts of Nepal, which were mostly urban, however these did represent some geographic heterogeneity across the country. Data was collected during the COVID-19 pandemic, thereby participants and their experiences may have differed from those seeking medical abortions at other times. We could hypothesize that COVID-19 would have acted as a barrier to seeking care or led to more women having unintended pregnancies due to gaps in reproductive care access.(20,21) Despite these limitations, this study provides insight into the experiences of women seeking abortions at pharmacies in Nepal, an understudied experience.”

12) In addition to the valuable insights gained from this study, it is recommended to incorporate a dedicated 'Conclusions' section. This section can serve as a crucial endpoint to succinctly summarize and discuss the main findings, drawing connections to the broader implications for policy, practice, and future research.

Thank you, we have now added a conclusion that reads

Although pharmacies not currently registered as legal providers of abortion pills, many women seek medication abortion from pharmacies in Nepal. We find that women who are below the recommended clinical gestational age for medication abortion pills are able to obtain pills at pharmacies along with information in Nepal. Many women who seek abortion services through pharmacies do so because such services are easy to access and pharmacies are sites recommended by family and friends. Expansion of the law to allow pharmacy distribution would increase accessibility for people who cannot easily access government certified health facility . A change in law would reflect current practice in the country whereby pharmacies are a common first stop in seeking healthcare services.”

Reviewer #2: Review for “Predictors and experiences with pharmacy abortion denial in Nepal”

I appreciated the opportunity to review this manuscript regarding data from a longitudinal study of women’s experiences seeking and using medical abortion pills from pharmacies in Nepal. The topic and findings are very timely, given the political climate surrounding abortion in Nepal and around the world. I have a few comments and questions to help clarify and strengthen the manuscript.

Thank you for these positive comments!

General Comments
• This manuscript discusses pharmacy provision of abortion services. Does this mean provision without a prescription, with a prescription, or either? It would be helpful if you were more direct in stating that the study concerns illegal, over-the-counter abortion pill provision, if that is the case. Particularly in the abstract, the language about that is vague.

We have edited the first sentence of the abstract to read “Abortion was legalized in Nepal in 2002; however, despite evidence of safety and quality provision of medical abortion pills by pharmacies in Nepal and elsewhere, is still not legal for pharmacists to provide medication abortion in Nepal.”

• The manuscript discusses biases influencing who is approved or denied the abortion medication, but the results suggest that the decision is based almost entirely on whether participants meet the 10-week guideline, indicating that there is very little bias occurring. An analysis of women who are eligible based on the 10-week guideline, but denied anyway, would be necessary to make assertions about biases. The current study only has 5 individuals out of 143 who meet that description.

This is an excellent point. We have added the following in the results, and again highlighted this point in the conclusion “The main factor predicting receipt of abortion was gestational age at the time of abortion seeking; thus, our findings are more indicative of factors predicting who seeks medical abortion before compared to after 10 weeks of gestation (the legal cut off at clinics).” And in the conclusion “Although pharmacies not currently registered as legal providers of abortion pills, many women seek medication abortion from pharmacies in Nepal. We find that women who are below the recommended clinical gestational age for medication abortion pills are able to obtain pills at pharmacies along with information in Nepal.”

We also make the point in the discussion that there is not evidence of bias in care in our study.

Introduction
• It would be helpful to see a clearer explanation regarding the legality/process of obtaining abortion pills from the pharmacy (Is it legal with a prescription from a physician/midwife? How often do pharmacies employ an auxiliary nurse-midwife? Do the women or the pharmacists face legal consequences if caught? If so, what are they?)

We have added the following to the introduction “Though common, off-label or over the counter sales of MA drugs at pharmacies are not legally permitted in Nepal. As per the policy – both providers and place to obtain abortion services needs to be approved by the government. Pharmacies in Nepal are operated by varied professionals including pharmacists, pharmacy assistants, and health workers who have received 48–72 hours orientation on pharmacy. Drug dispensing is also common among paramedics in Nepal (3). Since it is illegal to obtain abortion services including MA pills, both women and pharmacies can face legal consequences if caught.”

• Lines 117-122: If the word limit permits, it would be useful to provide more information about the conflicting Nepal studies regarding pharmacy assisted abortion, beyond the fact that they conflict (i.e., what were the methods/samples and specific findings?)

We have clarified that both of these were systematic reviews, however one focused only in Nepal and was more recent, and the other was a broader review of all studies globally on the topic.

Materials and Methods
• Were there only four eligible pharmacies or did you select four from a greater number?

We have added that this was from a list of 19 potential pharmacy sites to the methods

• I would be interested to know what steps were taken to protect pharmacists and women from legal repercussions for participating.

As was approved by the Nepal research council, all women and pharmacists provided consent, we ensured confidentiality and privacy of the data, they had a right to refuse to participate and participation was voluntary. The study team keeps confidential the names of the pharmacies involved in the study.

Results
• The logistic regression analysis is misleading, because it doesn’t show what it purports to show regarding biases.
o According to guidelines, the cut off for abortion pill access is 10 weeks pregnant. Based on the data from table 2, whether a woman meets this guideline appears to be, by far, the main factor in whether she is approved or denied the medication (100% of approved women are under 10 weeks pregnant, 84% of denied women were over 10 weeks pregnant, and 96% of women under 10 weeks were approved).
o In effect, this regression is basically a comparison of people under 10 weeks and people over 10 weeks pregnant at the time abortion is sought (with a few exceptions).
o If you wanted to do a logistic regression to look at biases among who is approved or denied, you would need to look at people who are eligible based on the 10-week guidelines only, and compare differences in who is approved vs who is denied among that group. Of course, that’s not possible with only a handful of participants under 10 weeks being denied.

This is such a great point, thank you. We have added some more language throughout the paper to reinforce this point (that gestational age was a main predictor of abortion denial).

Discussion
• Minor, but lines 287-288 state that “All who were under 10 weeks’ gestation” received medical abortion services, but it was actually 96% according to Table 2.

Thank you, edited to read “most”
